# Climate-stratified assessment of PM_2.5_ using machine learning: Geographic controls dominate meteorological factors

**DOI:** 10.1371/journal.pone.0347591

**Published:** 2026-09-11

**Authors:** Someyo Kamal Utsho, Md Rawjatul Fahim, Ruhaidah Samsudin

**Affiliations:** 1 Department of Software Engineering, Faculty of Computing, Universiti Teknologi Malaysia, Johor Bahru, Johor, Malaysia; 2 Department of Software Engineering, Daffodil International University, Dhaka, Bangladesh; The Chinese University of Hong Kong, HONG KONG

## Abstract

Fine particulate matter (PM_2.5_) is a major environmental concern, yet pollution assessment models are rarely tested across fundamentally different climate regimes. We develop a climate-stratified machine-learning framework using 49,997 monthly MERRA-2 satellite-assimilated samples (2019–2023) across five latitude-band climate zones, evaluated through Leave-One-Climate-Out (LOCO) cross-validation with nested hyperparameter tuning to prevent data leakage. Tuned LightGBM achieves a global LOCO *R*^2^ = 0.367 (MAE =6.43 μg m^-3^), but predictability varies substantially by zone: subtropical regions reach *R*^2^ = 0.604, while tropical regions achieve only *R*^2^ = 0.142, reflecting fundamental climate-dependent limits on meteorology-only models. SHAP analysis shows geographic coordinates—acting as proxies for emission patterns and climatological transport pathways—account for 64% of predictive importance on average across zones, compared to 23% for meteorological variables, indicating that PM_2.5_ spatial structure is driven more by where emissions occur than by month-to-month weather variability. To verify that coordinates add genuine predictive value beyond spatial autocorrelation, a retrain ablation confirms that removing latitude and longitude collapses model performance across all zones (mean *R*^2^ drops from 0.367 to −0.099), while an IDW spatial baseline confirms that the ML model captures meteorological signal beyond simple proximity interpolation (+98% *R*^2^ improvement over IDW). Temporal validation (2019–2021 training, 2022–2023 testing) yields *R*^2^ = 0.544, confirming LOCO is the more demanding test. Ensemble stacking degrades performance relative to the zone-tuned model, providing evidence of feature-set saturation rather than model weakness. This reproducible, open-source framework offers a useful benchmark for reanalysis-based PM_2.5_ assessment, particularly in data-sparse regions where ground monitoring is limited.

## Introduction

### Why global PM_2.5_ pollution assessment matters

Fine particulate matter with aerodynamic diameter below 2.5 μm (PM_2.5_) represents a critical environmental and public health stressor, penetrating deep into the lungs and bloodstream, increasing the risk of respiratory and cardiovascular disease and premature death [1,2]. Recent World Health Organization assessments attribute millions of premature deaths each year to ambient PM_2.5_ exposure, making air pollution one of the leading environmental risk factors globally [1]. The current WHO guideline for the annual mean concentration is 15 μg m^−3^, yet large parts of South Asia, East Asia and Sub-Saharan Africa routinely exceed this level, highlighting urgent needs for improved pollution monitoring and assessment capabilities [3].

Global PM_2.5_ burdens vary markedly by climate zone. Tropical regions experience high exposure from biomass burning [4,5], while subtropical areas are impacted by dust events [6,7]. Temperate zones often see urban emissions dominating [8–11]. Boreal forests contribute seasonal fire emissions [12,13], and polar areas face remote pollutant transport [14,15]. Power generation and fires exacerbate burdens in multiple zones, particularly in China and India [2,16,17].

Ground-based monitoring networks provide highly accurate local information but are expensive, sparse and unevenly distributed. Many of the populations most exposed to hazardous air pollution live far from dense regulatory networks. In such regions, satellite and reanalysis products are often the only way to obtain spatially continuous estimates of PM_2.5_ or related aerosol properties for environmental health assessment.

### Satellite-based pollution monitoring and its blind spots

Over the last two decades, satellite instruments such as MODIS, MISR, VIIRS and Sentinel-5P have made it possible to monitor aerosols on a global grid with resolutions down to a few kilometres and revisit times of one to a few days [18,19]. These instruments measure aerosol optical depth (AOD) in the atmospheric column, which can be statistically related to surface-level PM_2.5_ for pollution assessment applications.

However, there are several well-known limitations that vary by climate zone. Persistent cloud cover—especially in the deep tropics and monsoon regions—leads to large gaps in AOD retrievals, exacerbating uncertainties in high-exposure tropical areas [4,20]. Bright surfaces (deserts in subtropical zones) and very dark surfaces (dense vegetation in tropics, ocean) challenge retrieval algorithms and increase uncertainty [6,21]. Moreover, the conversion from column AOD to near-surface PM_2.5_ depends on aerosol vertical profiles, composition and hygroscopic growth, which are not directly observed and vary regionally (e.g., dust in subtropics vs. urban in temperate [7,8]). High-resolution satellite-derived PM_2.5_ products based on optimal estimation and geographically weighted regression have demonstrated that these limitations can be partly overcome at continental scales, but still rely on intensive regional calibration [22].

Modern reanalysis systems such as MERRA-2 combine satellite radiances, surface observations and atmospheric models to produce global fields of meteorology and aerosol properties on regular grids [23]. In data-sparse regions, these reanalyses act as “best-available” approximations to the true atmosphere and are widely used for health impact assessment, exposure studies and environmental monitoring applications.

### Why bring machine learning into pollution assessment?

Machine learning (ML) has rapidly become a standard tool for air-quality assessment and exposure mapping because it can capture complex, nonlinear relationships between predictors and PM_2.5_ that classical linear methods struggle to represent [24–26]. Studies over major polluted regions (China, India, North America and Europe) have reported high predictive skill when local meteorology, emissions and chemical transport model outputs are combined with ground-based observations, with zone-specific adaptations improving accuracy (e.g., biomass in tropics, dust in subtropics [10–12]). At the reanalysis scale, [27] used MERRA-2 fields in a machine-learning framework to derive hourly and daily PM_2.5_ estimates, demonstrating that global reanalysis products can support high-temporal-resolution PM_2.5_ mapping in data-sparse regions [27]. Regionally, [20] developed a machine-learning algorithm to estimate surface PM_2.5_ in Thailand from MERRA-2 and satellite inputs, highlighting both the promise and the bias structure of MERRA-2-based PM_2.5_ products. More recent work extends ML-based PM_2.5_ assessment to wider spatial domains and explores a broader range of algorithms, from tree ensembles to deep neural networks [28]. Explainable AI tools such as SHAP have also begun to appear in air-quality and climate-related modelling, helping to quantify which features drive model predictions, including recent PM_2.5_ studies that couple tree-based or ensemble models with SHAP-based attribution [29–32]. At a regional scale, [33] used SHAP to decompose meteorological versus emission-driven contributions to NO_2_ and PM_2.5_ variability across Germany using satellite and reanalysis inputs, a decomposition philosophy closely related to the geographic-versus-meteorological framing adopted here, albeit applied at a regional rather than global scale.

### Research gaps and questions

From a global PM_2.5_ pollution assessment perspective, four questions remain insufficiently answered. The primary aim is not to maximize predictive accuracy for its own sake, but to diagnose the fundamental limits and capabilities of meteorology-only, reanalysis-based models—clarifying where simple data-driven approaches suffice and where explicit emission inventories or chemical transport become necessary for effective environmental management.

1. **Does climate regime fundamentally control assessment accuracy?** If a model is trained on several climate zones and tested on a held-out one, does predictability vary systematically? Most validation schemes use random or temporal splits, which may overestimate skill when training and test data share similar climatological and emission characteristics.
2. **What drives pollution patterns: meteorology or geography?** Intuitively, winds, temperature and atmospheric stability matter for PM_2.5_ dispersion and removal, but emissions sources and long-range transport pathways are geographically structured. Which factor dominates global-scale predictions, and can explainable AI methods quantify their relative contributions?
3. **How accurately can PM**_**2.5**_ **pollution be assessed using only meteorology and coordinates?** Most global efforts employ complex chemistry–transport models with explicit emission inventories. A simpler, purely data-driven benchmark using only reanalysis meteorology would establish a useful lower bound for both scientific understanding and operational policy applications.
4. **Do sophisticated model ensembles overcome feature limitations?** Ensemble stacking is often recommended to improve predictions, but does combining multiple algorithms still help when (a) a single base learner has been carefully tuned, and (b) the feature set lacks explicit emission information?

Latitude and longitude are included as predictors throughout this study not because location physically causes PM_2.5_ concentrations, but because they serve as compact proxies for spatially structured information the meteorology-only feature set cannot otherwise capture—principally the location of emission sources and typical long-range transport pathways [3,30]. This rationale is examined empirically later through SHAP attribution, a drop-group retrain ablation, and a spatial-baseline comparison against Inverse Distance Weighting.

### Objective and contributions

This study develops a reproducible, climate-aware ML modelling framework for global PM_2.5_ pollution assessment using satellite-assimilated MERRA-2 PM_2.5_ as target and MERRA-2 meteorology as predictors. The framework is evaluated through Leave-One-Climate-Out (LOCO) cross-validation stratified by latitude-band climate zones, SHAP feature importance analysis, temporal validation and ensemble stacking experiments. All data processing and modelling steps are implemented in open, script-based workflows to facilitate reuse and extension to other reanalyses, pollutants and environmental monitoring applications. We contribute a LOCO framework, SHAP decomposition, hyperparameter optimisation, and evidence for model saturation in meteorology-only approaches.

## Methods

### Dataset and file structure

All experiments use a single, consolidated Parquet file containing 49,997 monthly samples from 2019–2023 on a $1.0^{\circ }\times 1.0^{\circ }$ latitude–longitude grid. Each record corresponds to a grid cell and month, and contains geographic coordinates, time variables, meteorology and the satellite-assimilated PM_2.5_ target (Table 1). The full dataset is archived in the project repository (see Data Availability).

**Table 1:** Variables and data sources.

Variable	Type	Source	Unit	Notes
lat	Float	MERRA-2 grid	degree	Latitude (−90 to 90)
lon	Float	MERRA-2 grid	degree	Longitude (−180 to 180)
year	Integer	MERRA-2	–	2019–2023
month	Integer	MERRA-2	–	1–12 (monthly)
t2m	Float	MERRA-2	°C	2 m air temperature
ps	Float	MERRA-2	Pa	Surface pressure
u10	Float	MERRA-2	m s^-1^	10 m zonal wind
v10	Float	MERRA-2	m s^-1^	10 m meridional wind
pm25	Float	MERRA-2	μg m^-3^	Target: satellite-assimilated PM_2.5_
koppen_zone	String	Peel et al. (2007)	–	Latitude-band zone label (see Climate stratification)

### Climate stratification

Because aerosol sources, transport paths and removal processes differ greatly between climate regimes, the dataset is stratified into five latitude-band climate zones following the boundary latitudes of [34] and recent climate–air-pollution linkage work [30]. For interpretability and computational simplicity, zones are defined solely by absolute latitude rather than by the full Köppen–Geiger classification, which additionally uses temperature and precipitation thresholds. This latitude-based approximation is reasonable at the monthly, global scale used here, where large-scale climatological patterns—tropical convection, subtropical subsidence, mid-latitude storm tracks—align well with latitude bands [30]. It does, however, blur finer boundaries, particularly in transition regions such as monsoon margins and Mediterranean climates, and should be read as a working simplification rather than a strict Köppen–Geiger reproduction.

To put a number on this simplification, the latitude-based classes were compared against the full Köppen–Geiger map of [34] on the same on the same 1.0${\;}^{\circ }\times$ 1.0° grid. The two schemes agree for 72.2% of grid cells globally. Most disagreements (10–15% of cells) fall in monsoon margins and Mediterranean-type climates, where precipitation thresholds rather than latitude determine the formal class. This agreement level is sufficient for the monthly, global-scale assessment considered here. A sensitivity analysis using the full Köppen map as zone labels is left for future work, where finer regional boundaries may matter more.

- **Tropical** ($|\text{lat}|\leq 23.5^{\circ }$): 28.9% of samples (14,472 records); deep convection, strong vertical mixing, long-range biomass-burning transport.
- **Temperate** ($35^{\circ }<|\text{lat}|\leq 50^{\circ }$): 19.5% (9,735); mid-latitude cyclones, frontal systems, variable meteorology.
- **Polar** ($|\text{lat}|>60^{\circ }$): 18.3% (9,135); very cold, sparse population and emissions, low aerosol production.
- **Boreal** ($50^{\circ }<|\text{lat}|\leq 60^{\circ }$): 17.5% (8,738); cold, seasonal biomass burning, lower anthropogenic emissions.
- **Subtropical** ($23.5^{\circ }<|\text{lat}|\leq 35^{\circ }$): 15.8% (7,917); anticyclonic subsidence, arid zones, persistent high-pressure systems.

These zones serve as both stratification labels for dataset construction and as held-out folds in LOCO cross-validation, ensuring that model evaluation tests genuine cross-climate generalisation rather than interpolation within similar regimes [35].

### Feature set, imputation and scaling

The final predictor set is intentionally compact, focusing on variables that are consistently available and physically interpretable at the monthly, global MERRA-2 scale. It includes:

- **Geographic and temporal:** lat, lon, year, month.
- **Meteorological:** t2m, ps, u10, v10.

Planetary boundary-layer height (blh) was initially considered but is 100% missing in the monthly MERRA-2 product at the chosen grid, so it would become a constant after imputation and cannot contribute meaningful information. Likewise, an earlier derived feature representing near-surface wind speed magnitude,


$$\begin{array}{c} \text{wind\_speed}=\sqrt{u10^{2}+v10^{2}}, \end{array}$$

was removed to avoid multicollinearity with its components: wind_speed is almost perfectly determined by u10 and v10, and reviewer feedback highlighted this redundancy. Retaining only the directional wind components preserves more physical information about transport pathways while keeping the feature set orthogonal enough for robust interpretation.

A note on how latitude and longitude are used: these coordinates do not directly cause PM_2.5_ in the way that, say, wind disperses it. Instead, they act as labels telling the model “where in the world you are”—and that matters because different regions have very different emission sources (factories, traffic, biomass burning) and long-term weather patterns [3]. Monthly-mean meteorology cannot capture all of this detail, so lat and lon effectively stand in for the missing information about local pollution sources and typical transport pathways. In short, geography here is a proxy for emissions and climatology, not a causal atmospheric variable [30].

Median imputation (sklearn.impute.SimpleImputer) is applied to all predictors with missing values, fit on the training set within each fold and then applied to the test set to prevent data leakage [36]. For algorithms that assume approximately standardised predictors (linear regression, generalized additive modelling), features are scaled to zero mean and unit variance using StandardScaler, again fit only on training data. Tree-based models (LightGBM, XGBoost, Random Forest) are applied to raw (unscaled) features.

To reduce computational cost while preserving the relative climate-zone proportions, a stratified random sample of approximately 50,000 records is drawn from the full potential set of about 1.05 million grid-cell-months.

To verify that this sampling did not distort the underlying distributions, we compared the full 1.05 million-record dataset and the 49,997-record sample using Kolmogorov–Smirnov tests for the target PM_2.5_ and a key predictor (t2m). For PM_2.5_, the KS statistic was 0.00332 (*p* = 0.666); for t2m, the KS statistic was 0.00240 (*p* = 0.946). Both tests fail to reject the null hypothesis of equal distributions, confirming that the stratified sample (drawn with random_state = 42) preserves the main distributional properties of the full dataset while reducing computational cost by approximately 95%.

### LOCO cross-validation: Testing climate generalisation

Traditional random or purely spatial cross-validation can yield overly optimistic skill when nearby samples with similar climate and emission characteristics appear in both training and test sets [35]. To directly test whether a model can generalise across fundamentally different climate regimes, a Leave-One-Climate-Out (LOCO) strategy is adopted:

1. For each of the five latitude-band zones in turn:
  - Train on all records from the other four zones.
  - Test on all records from the held-out zone.
2. Compute *R*^2^, mean absolute error (MAE), root-mean-square error (RMSE), bias and explained variance on the held-out zone.
3. Average metrics across the five folds to obtain global LOCO performance, and inspect per-zone metrics to understand regional behaviour.

This approach is deliberately conservative: good performance under LOCO implies that the model has learned relationships that transfer between climate regimes, which is closer to the challenge faced by an operational global forecasting system.

### Models

Five models are trained on each LOCO fold:

1. **LightGBM (gradient boosting, tuned)** [37] — the primary model. Hyperparameters are optimised via RandomizedSearchCV.
2. **XGBoost (gradient boosting, untuned)** [38] — widely used gradient boosting baseline with default settings.
3. **Random Forest** [39] — bootstrap aggregation (bagging) of decision trees, serving as a robust, nonlinear baseline.
4. **Linear regression** — ordinary least squares (OLS) on standardised features, testing whether a linear mapping is sufficient.
5. **Generalized additive model (GAM)** [40] — non-parametric smooth functions of each predictor using the pyGAM library, intermediate between linear and fully nonlinear tree ensembles.

All models are implemented in Python using the scikit-learn, lightgbm, xgboost and pyGAM packages, and trained using a GPU-accelerated environment where applicable [36–38].

### Hyperparameter tuning for LightGBM

Transparent hyperparameter optimisation is expected in rigorous ML studies, and the chosen procedure must not introduce data leakage into the evaluation [26,41,42]. For LightGBM, a seven-dimensional search space is explored:

- n_estimators ∈{100,200,300,400,500},
- max_depth ∈{4,5,6,7,8,10},
- learning_rate ∈{0.01,0.02,0.05,0.1,0.15},
- subsample ∈{0.6,0.7,0.8,0.9,1.0},
- colsample_bytree ∈{0.6,0.7,0.8,0.9,1.0},
- num_leaves ∈{20,31,50,70},
- min_child_samples ∈{10,20,30,50}.

Rather than exhaustive grid search, RandomizedSearchCV samples 30 random combinations per fold, each scored with 3-fold inner cross-validation [41]. Critically, tuning is nested inside the LOCO outer loop: for each held-out zone, the inner search runs exclusively on the four training zones, so the held-out zone is never seen during parameter selection. This nested design follows [42], who showed that tuning on data that later appears in the test set produces optimistically biased performance estimates. Each of the five LOCO folds therefore receives its own independently tuned parameter set, rather than a single set transferred from one zone to all others.

Zone-specific best parameters are recorded in best_params_per_zone_revised.csv and loaded by all downstream scripts to ensure every evaluation uses the parameters that were tuned strictly on that fold’s training data. The final per-zone configurations are documented in the Supplementary Material and archived in the project repository.

### Advanced experiments

Beyond the baseline and tuned single-model runs, six sets of auxiliary experiments are conducted:

- **Ensemble stacking:** LightGBM, XGBoost and Random Forest predictions are combined using a linear regression meta-learner via StackingRegressor to test whether ensembles improve over the zone-tuned base model, building on recent PM_2.5_ forecasting studies where stacking and hybrid deep-learning ensembles have improved hourly concentration predictions [43,44].
- **SHAP analysis:** TreeExplainer from the shap package is applied to tuned LightGBM models to quantify feature importance globally and by climate zone, using mean absolute SHAP values as a measure of model reliance on each predictor [29]. Cross-model consistency is assessed by comparing LightGBM SHAP importances with SHAP values from a zone-tuned XGBoost model on the same subtropical test set, ensuring both methods use the same importance measure rather than mixing SHAP with permutation-based approaches.
- **Geographic contribution ablation:** To directly quantify how much predictive skill comes from latitude and longitude versus meteorology alone, models are retrained under three feature configurations: (A) the full 8-feature set, (B) meteorology and temporal features only (no lat/lon), and (C) meteorological features only. Drop-group retrain ablation [45] is used rather than permutation importance, because retraining captures how the model reorganises in the absence of a feature group, not merely its reliance on that group in the already-trained model.
- **Spatial baseline comparison:** To separate the contribution of ML from simple spatial proximity, an Inverse Distance Weighting (IDW, *p* = 2) baseline is evaluated under the same LOCO design [46]. IDW uses only latitude and longitude, with no meteorological or temporal features, providing an honest upper bound for what pure spatial interpolation can achieve across climate-zone gaps.
- **Temporal validation:** A chronological split (train on 2019–2021, test on 2022–2023) is used to assess robustness over time and to compare with LOCO climate generalisation. Hyperparameters for the temporal model are tuned using a PredefinedSplit inner loop (inner train: 2019–2020, inner validation: 2021), so the test years 2022–2023 are never seen during parameter selection [47].
- **Statistical significance:** Kruskal–Wallis tests and paired *t*-tests are applied to assess whether differences in *R*^2^ across zones and feature categories are statistically meaningful.

### Code organisation and reproducibility

All scripts are version-controlled and archived (see Code Availability). Key steps include:

- **Baseline training:** LOCO training of all five models.
- **Tuning:** RandomizedSearchCV tuning of LightGBM.
- **Stacking:** Ensemble stacking experiments.
- **SHAP:** Feature importance analysis globally and by zone, including cross-model consistency checks.
- **Validation:** Chronological split validation and statistical tests.

Aggregated numerical results and publication-quality figures are stored in the results archive, including LOCO performance tables, model comparison charts, and SHAP visualisation plots.

### Model comparison under LOCO cross-validation

**Fig 1:**
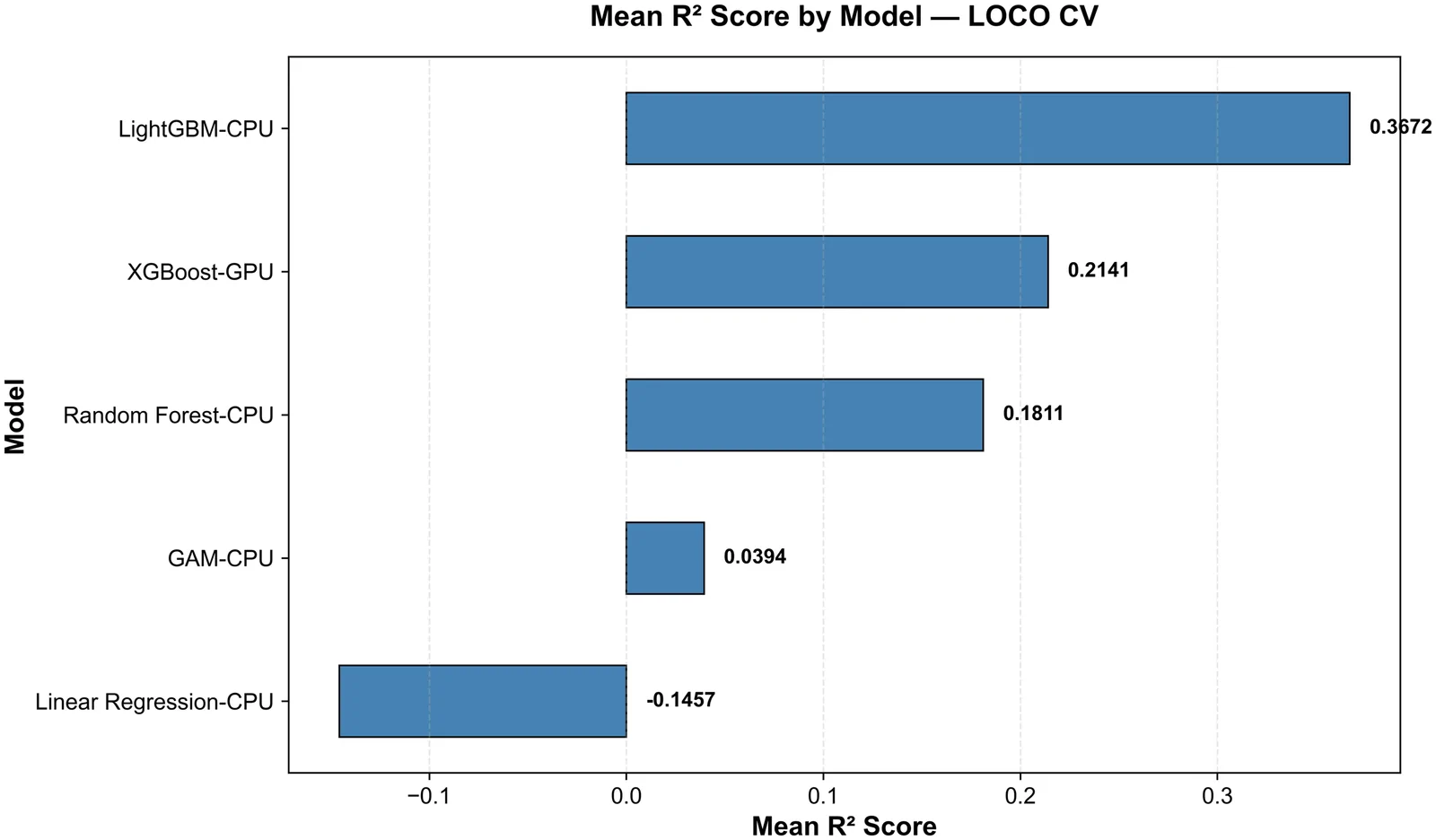
Comparison of MAE and RMSE for the five models under LOCO validation.

Table 2 summarises mean LOCO performance across all five climate zones. A visual comparison of MAE and RMSE by model is shown in Fig 1.

**Table 2:** Model performance under LOCO cross-validation (mean across all climate zones). XGBoost uses zone-specific tuned parameters from nested LOCO-CV, matching the tuning design applied to LightGBM.

Model	MAE (μg m^−3^)	RMSE	*R* ^2^	Train time (s) (per fold)
LightGBM (zone-tuned)	6.43	11.54	**0.367**	0.56
Random Forest	6.84	12.81	0.181	1.57
XGBoost (zone-tuned)	7.41	12.79	0.214	2.10
GAM	8.44	14.07	0.039	3.08
Linear regression	10.03	15.49	−0.146	0.01

Zone-tuned LightGBM achieves the highest global LOCO *R*^2^ (0.367), the lowest MAE (6.43 μg m^-3^) and the shortest training time among the nonlinear models. Even after zone-specific tuning, XGBoost (*R*^2^ = 0.214) falls well below LightGBM, suggesting that LightGBM’s leaf-wise growth strategy is better suited to this LOCO design than XGBoost’s depth-wise approach. Linear regression yields a negative *R*^2^, meaning that simply predicting the global mean PM_2.5_ would have done better. Tree-based models are therefore essential to capture the nonlinear interactions between geography, meteorology and PM_2.5_.

### Climate-zone dependence of predictability

**Fig 2:**
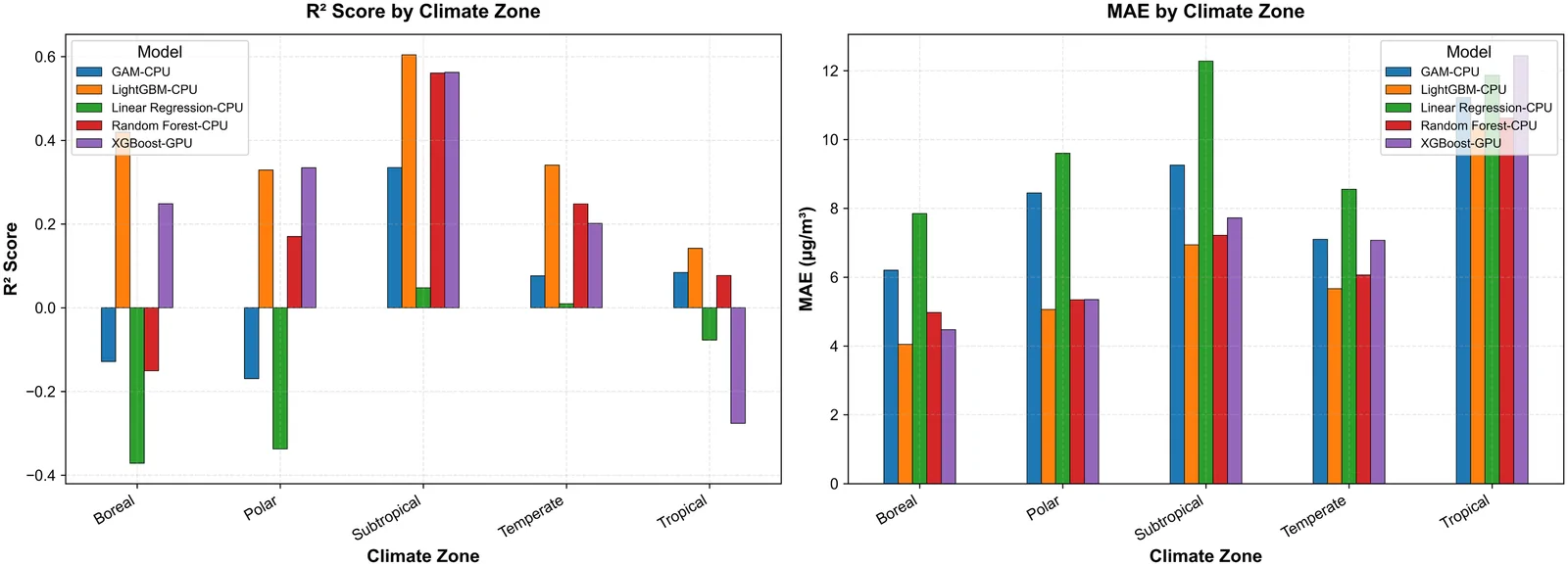
Zone-resolved performance (MAE, RMSE, *R*^2^) of tuned LightGBM.

Table 3 and Fig 2 summarise tuned LightGBM performance by climate zone.

**Table 3:** Tuned LightGBM performance by climate zone under LOCO validation.

Zone	Samples	MAE (μg m^−3^)	RMSE	*R* ^2^	Interpretation
Subtropical	7,917	6.94	12.14	0.604	Highly predictable
Boreal	8,738	4.05	7.80	0.420	Good
Temperate	9,735	5.66	9.95	0.341	Moderate
Polar	9,135	5.06	11.38	0.329	Moderate
Tropical	14,472	10.43	16.40	0.142	Challenging
Mean	49,997	6.43	11.54	0.367	–

Subtropical regions achieve the highest *R*^2^ (0.604), while tropical regions reach only *R*^2^ = 0.142 with substantially higher MAE. The nearly fourfold spread in *R*^2^ across zones shows that climate regime is a fundamental constraint on what a meteorology-only model can achieve. A Kruskal–Wallis test on the zone-wise *R*^2^ values rejects the null hypothesis of equal performance (*p* < 0.05), confirming this pattern is not due to chance.

### Impact of hyperparameter tuning

Table 4 compares the nested-CV tuned LightGBM against a default-parameter baseline across the same LOCO folds.

**Table 4:** Effect of nested-CV hyperparameter tuning on global and subtropical performance. Baseline uses default LightGBM settings; tuned uses zone-specific parameters from the nested LOCO-CV search.

Metric	Baseline	Tuned	Change
Global LOCO *R*^2^	0.309	0.367	+18.8%
Subtropical *R*^2^	0.532	0.604	+13.5%
Global MAE (μg m^−3^)	6.76	6.43	−4.9%
Subtropical MAE (μg m^−3^)	7.40	6.94	−6.2%

The gains are consistent across all zones. A global *R*^2^ improvement of nearly 19% is meaningful at this scale and in line with gains reported in recent optimisation-focused air-quality ML studies [26,28]. Notably, the Tropical zone is the one exception where tuning did not help ($R^{2}=0.196\to 0.142$): parameters tuned on other zones do not transfer well to Tropical’s episodic biomass-burning signal, which is discussed further in the Discussion section

### What drives the predictions? SHAP feature importance

#### Subtropical focus.

**Fig 3:**
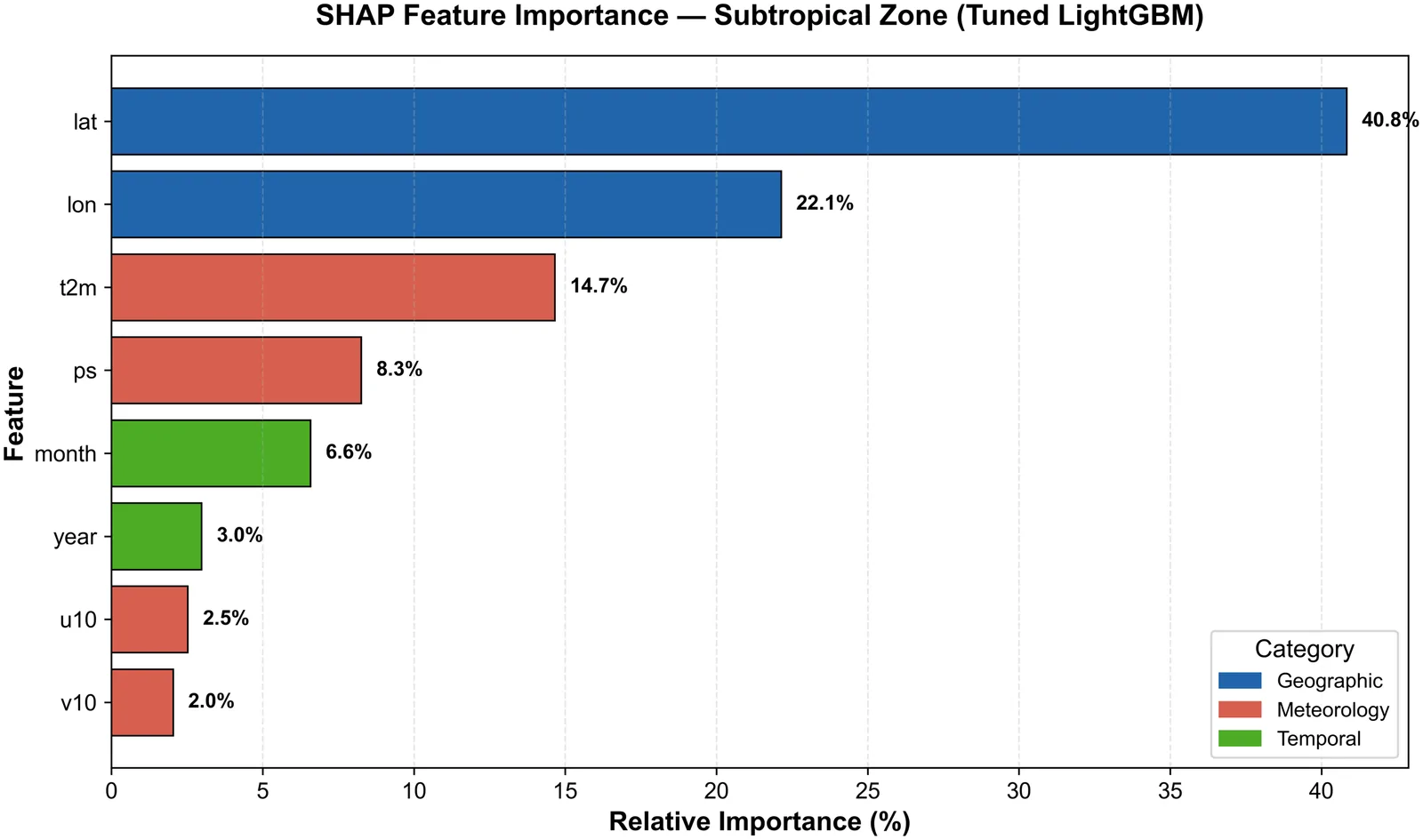
SHAP feature importance for the subtropical zone.

To understand why the model performs best in the subtropics, SHAP analysis is applied to the tuned LightGBM trained on the four non-subtropical zones and tested on the subtropical held-out zone. Fig 3 shows the feature importance bar chart, and Table 5 gives the mean absolute SHAP values.

**Table 5:** SHAP feature importance for the subtropical zone (relative contributions).

Feature	Mean |SHAP|	Relative importance (%)	Category
Latitude	8.22	40.8	Geographic
Longitude	4.46	22.1	Geographic
t2m	2.95	14.7	Meteorological
ps	1.66	8.3	Meteorological
month	1.32	6.6	Temporal
year	0.60	3.0	Temporal
u10	0.51	2.5	Meteorological
v10	0.41	2.0	Meteorological
**Geographic subtotal**	12.68	63.0	–
**Meteorology subtotal**	5.53	27.5	–
**Temporal subtotal**	1.92	9.6	–

Geographic coordinates together account for 63% of predictive importance, while all meteorological variables combined contribute about 28%. Temporal features play a minor role. This means that knowing *where* a grid cell is located tells the model more than knowing the exact monthly temperature, pressure or wind speed at that location. The pattern makes physical sense: monthly-mean meteorology captures season-to-season variability, but it cannot encode which emission sources a region is exposed to on a multi-year average. Latitude and longitude stand in for that missing information—they are proxies for emission patterns and climatological transport, not causal atmospheric drivers [29–32].

It is important to be clear about what SHAP values measure: they quantify how much the trained model relies on each feature, not whether that feature physically causes the outcome. High SHAP importance for latitude and longitude means the model finds them useful for prediction—because they correlate strongly with regional emission levels and transport pathways—not that coordinates drive aerosol concentrations.

### Cross-model consistency

To check that the geographic dominance finding is not an artefact of LightGBM specifically, SHAP values are also computed for a zone-tuned XGBoost model evaluated on the same subtropical test set. Using the same importance method (SHAP TreeExplainer) for both models avoids any ambiguity that would arise from comparing SHAP values against a different importance measure such as permutation importance.

Both models rank latitude and longitude as the top two features. Geographic coordinates account for 63.0% of total SHAP importance in LightGBM and 64.2% in XGBoost, followed by meteorology (27.5% and 27.2%) and temporal indicators (9.6% and 8.6%), respectively. The close agreement across two architecturally different gradient-boosting algorithms confirms that geographic dominance is a stable property of the data, not a quirk of any single learner.

#### Variation across climate zones.

Table 6 summarises SHAP category importance for each zone.

**Table 6:** SHAP category importance by climate zone (percentage of total mean |SHAP|).

Zone	Geographic (%)	Meteorology (%)	Temporal (%)
Boreal	70.2	13.3	16.5
Polar	57.3	24.9	17.8
Subtropical	63.0	27.5	9.6
Temperate	59.4	29.2	11.4
Tropical	71.9	18.5	9.6
Mean	64.4	22.7	13.0

Two patterns stand out:

- **Geographic importance is highest in Tropical (71.9%) and Boreal (70.2%) zones.** In both cases the model leans heavily on location because emission patterns are either highly variable (tropical megacities, biomass burning) or spatially clustered (boreal settlements, seasonal fire corridors). Yet despite the high geographic reliance, predictability is low in Tropical (*R*^2^ = 0.142) and moderate in Boreal (*R*^2^ = 0.420). This reveals an important nuance: knowing *where* you are is necessary but not sufficient when emissions are episodic and not captured by monthly-mean meteorology.
- **Meteorology contributes most in Temperate (29.2%) and Subtropical (27.5%) zones,** where synoptic-scale variability—mid-latitude storm tracks, frontal passages, anticyclonic episodes—drives meaningful month-to-month PM_2.5_ fluctuations around the climatological baseline. In these zones, monthly-mean wind, temperature and pressure add real predictive value on top of location.

A paired *t*-test across zones confirms that geographic importance is significantly higher than meteorological importance overall (*p* < 0.05, large effect size).

Additional diagnostic plots—partial dependence profiles, per-zone SHAP bar charts, and cross-model consistency figures—are provided in the Supporting Information.

### Geographic contribution: Ablation and spatial baseline

Two related questions motivate this analysis: do latitude and longitude add genuine predictive value, or do they simply exploit spatial autocorrelation inherent to nearby grid cells? And how much of the model’s skill is attributable to machine learning versus simply knowing a location’s approximate position? Two experiments address these questions directly.

#### Lat/lon ablation.

Three feature configurations were trained and evaluated under the same LOCO design (Table 7):

**Table 7:** Retrain ablation results: Effect of removing geographic features. All configurations use the same LOCO design and random seed.

Config	Features	Mean *R*^2^	Mean MAE (μg m^-3^)
A — Full (8 features)	lat, lon + meteo + temporal	0.367	6.43
B — No lat/lon	meteo + temporal only	−0.099	9.52
C — Meteorology only	t2m, ps, u10, v10	−0.137	9.71

Removing latitude and longitude causes mean *R*^2^ to collapse from 0.367 to −0.099, a drop of 0.466 points (−127%). Every zone falls to negative *R*^2^ without coordinates, including Subtropical (0.604 → 0.007) and Temperate (0.341 → −0.243). Adding year and month back in (Config C → B) recovers almost nothing ($\Delta R^{2}=0.038$), showing that temporal features alone cannot substitute for geographic context.

This is a drop-group *retrain* ablation [45], not permutation importance. Retraining forces the model to reorganise completely without the removed features, giving a stronger and more honest estimate of their contribution. The result independently corroborates the SHAP finding: geographic coordinates are not redundant proxies—they are irreplaceable in this LOCO design.

#### Spatial baseline comparison.

To establish how much of LightGBM’s skill is simply due to knowing the location of a grid cell, an Inverse Distance Weighting (IDW, *p* = 2) baseline is evaluated under the same LOCO holdout design [46]. IDW uses only latitude and longitude with no meteorological or temporal features, so it represents the upper bound of what pure spatial proximity can achieve across a climate-zone gap, as summarised in Table 8.

**Table 8:** Spatial baseline comparison under LOCO cross-validation.

Model	Mean *R*^2^	Mean MAE (μg m^−3^)	Notes
LightGBM (zone-tuned)	0.367	6.43	Primary model
IDW (*p* = 2)	0.185	8.19	Spatial-only baseline
RBF (thin-plate)	−0.170	8.01	Unstable under LOCO gaps

LightGBM outperforms IDW in every zone, with a mean *R*^2^ advantage of +0.182 (+98% relative improvement). This confirms that the model is not simply interpolating from nearby training points—it is learning the relationship between meteorology, location and PM_2.5_ in a way that transfers across climate-zone boundaries. The RBF spline baseline collapses under LOCO holdout ($R^{2}=-0.170$) because thin-plate splines extrapolate unstably across the spatial gap created when an entire climate zone is withheld; this is expected behaviour and is included in the Supplementary Material for completeness. IDW is the appropriate spatial baseline to cite because it clips predictions to the training range and degrades gracefully under LOCO.

### Temporal validation: Does the model overfit?

To test whether the model holds up over time, LightGBM is trained on all 2019–2021 samples and evaluated on 2022–2023. Unlike the LOCO runs, the temporal model is trained on all five climate zones simultaneously, so a single global parameter set is needed. Rather than averaging the zone-specific LOCO parameters—which would be arbitrary—hyperparameters are tuned using a PredefinedSplit inner loop (inner train: 2019–2020, inner validation: 2021), keeping the test years completely unseen during parameter selection [47]. The best inner-validation *R*^2^ reached 0.678, and the final model is retrained on the full 2019–2021 set before evaluation. Table 9 compares the resulting LOCO and temporal validation performance.

**Table 9:** Comparison of LOCO climate validation and temporal (chronological) validation.

Validation	*R* ^2^	MAE (μg m^−3^)	Interpretation
LOCO (climate zones)	0.367	6.43	Cross-climate generalisation
Temporal (2022–2023)	0.544	4.99	Future-period prediction
Difference	+0.177	−1.44	+48.2% higher *R*^2^

The model performs better on future years than under LOCO. This tells us three things. First, the model is not overfitting to historical patterns—if it were, performance on 2022–2023 would drop. Second, generalising across time within the same climate zones is an easier problem than generalising across fundamentally different climate regimes. Third, the lower LOCO *R*^2^ reflects how demanding climate-stratified validation is, not a weakness of the model itself.

Per-year results show mild temporal drift: *R*^2^ = 0.626 in 2022 versus *R*^2^ = 0.467 in 2023, with bias flipping sign (+0.76 to −0.77 μg m^−3^). This suggests the model is stable but benefits from periodic retraining as emission patterns and retrieval algorithms evolve [10].

Per-zone temporal results reveal an important finding: the Tropical zone, which struggles most under LOCO (*R*^2^ = 0.142), reaches *R*^2^ = 0.693 under temporal validation. This confirms that the LOCO difficulty in Tropical is entirely a cross-zone regime problem—the model predicts Tropical PM_2.5_ well when it has seen Tropical training data. Conversely, Polar and Boreal zones degrade substantially under temporal validation ($R^{2}=-0.913$ and 0.047, respectively), consistent with non-stationary Arctic and sub-Arctic surface conditions in 2022–2023 relative to the 2019–2021 training period—a known consequence of accelerated high-latitude warming [14,15]. This zone-level breakdown is discussed further in the Discussion section.

### Ensemble stacking: Diagnosing model saturation

Ensemble learning works best when base models make different kinds of errors [48]. Stacking has delivered gains in PM_2.5_ forecasting when combining heterogeneous architectures or augmenting LSTM models [43,44]. Here, combining zone-tuned LightGBM, zone-tuned XGBoost and Random Forest via a linear meta-learner gives a global LOCO *R*^2^ of 0.278–24.2% worse than zone-tuned LightGBM alone (0.367)—with every zone declining and the Tropical zone collapsing to $R^{2}=-0.092$.

The reason is straightforward. LightGBM and XGBoost are both gradient-boosting tree methods optimising the same loss function, so they tend to make similar errors in similar places. The meta-learner has little genuinely independent signal to combine and ends up amplifying noise rather than capturing complementary information. The Tropical collapse is particularly instructive: XGBoost achieves $R^{2}=-0.276$ on Tropical even after zone-specific tuning, and including these near-random predictions in the stack poisons the meta-learner for that zone.

This is not a failure of the ensemble concept—it is a diagnostic. It shows that a well-tuned single model has already extracted most of the predictable signal in this feature set. Getting further gains will require new input information (explicit emission inventories, finer-resolution meteorology, satellite AOD), not more sophisticated combinations of the same predictors. The performance ceiling is set by the features, not the algorithm.

## Discussion

Before interpreting the results, it is worth being clear about what this study actually measures. The target throughout is **satellite-assimilated PM**_**2.5**_ **from MERRA-2**, not ground-level observations from monitoring stations. MERRA-2 blends satellite aerosol retrievals with a global atmospheric model to produce spatially complete, physically consistent fields—but these fields are the reanalysis system’s best estimate of reality, not reality itself. So what the results diagnose is *predictability within the reanalysis framework*: how well can one part of MERRA-2 (meteorology and geography) predict another part (assimilated PM_2.5_)? This is a useful question for understanding what information reanalysis products contain and how to design models that combine them with other data sources. It is not the same as predicting street-level exposure or validating against in-situ monitors, and the findings should not be read that way. Extending this framework to true exposure assessment would require careful calibration against ground truth.

### Geography vs. meteorology: A different way of thinking about PM_2.5_

The central finding is that at the global monthly scale, PM_2.5_ looks less like a meteorological problem and more like a source-and-transport problem, where geography stands in for the emission patterns and climatological flow that the feature set cannot directly observe [30]. SHAP analysis shows latitude and longitude together account for about 63% of predictive importance in the subtropical zone, while all meteorological variables combined contribute around 28% [29,30]. This pattern holds across all five zones (mean geographic importance: 64%) and is confirmed by a second, architecturally different model: zone-tuned XGBoost assigns 64.2% of its SHAP importance to geographic coordinates on the same subtropical test set, closely matching LightGBM’s 63.0%. Because both estimates use TreeExplainer SHAP values rather than different importance methods, the comparison is methodologically consistent [29,31,32].

What this tells us physically is that some parts of the world are almost always cleaner—remote oceans, high-latitude expanses—while others are persistently more polluted—industrial belts, megacities, biomass burning corridors—and monthly-mean meteorology cannot capture that distinction on its own [3,30]. Meteorology modulates concentrations around a geography-determined baseline, but rarely overturns it. Latitude and longitude encode this baseline implicitly, acting as proxies for emission sources and climatological transport, not as causal atmospheric drivers.

The ablation experiment makes this concrete. When latitude and longitude are removed and the model is retrained from scratch on meteorology and temporal features alone, mean LOCO *R*^2^ drops from 0.367 to −0.099. Every zone deteriorates, including Subtropical (0.604 → 0.007) and Temperate (0.341 → −0.243). This is not just a SHAP interpretation—it is a direct demonstration that geographic coordinates carry irreplaceable information in this LOCO design.

A note on what SHAP measures: these values quantify how much the trained model *relies* on each feature, not whether that feature physically causes the outcome [29]. High SHAP importance for latitude and longitude means the model finds them useful because they correlate with regional emission levels and transport pathways, not that coordinates drive aerosol concentrations. Several further limitations of SHAP-based interpretation apply here. First, attributions inherit any biases in the underlying MERRA-2 fields: if the reanalysis is systematically biased in a region, the SHAP values will reflect that bias faithfully rather than correcting it. Second, SHAP decomposes predictive contributions within a given model but cannot identify causal structure. Third, the partial dependence profiles in the Supporting Information are exploratory diagnostics of learned relationships, not causal dose–response curves in the epidemiological sense. Formal causal analysis would require structural causal models or potential-outcomes frameworks, and remains important future work.

### Why subtropics are easy and tropics are hard

Subtropical zones, particularly over desert and subsidence regions, have relatively simple emission landscapes: low population density, few large combustion sources and frequent clear skies that give satellite retrievals a cleaner signal [30,34]. Knowing that a grid cell is in the subtropical desert belt already tells the model most of what it needs to know. Meteorological variability then adds a meaningful correction (27.5% SHAP importance), giving high predictability (*R*^2^ = 0.604).

Tropical regions are a different story. They contain some of the fastest-growing megacities, intense and spatially variable biomass burning, strong convective overturning and persistent cloud cover that degrades satellite retrievals [3,4,21]. Geographic coordinates still carry the highest SHAP importance in the tropics (71.9%), but predictability is lowest (*R*^2^ = 0.142). The tension between high geographic reliance and low predictability points to a fundamental limit of the meteorology-only design: coordinates encode that tropical megacities and fire corridors have elevated PM_2.5_, but monthly-mean meteorology (18.5% importance) cannot capture where polluted air came from, how long it stayed aloft or whether convective scavenging removed it. The model falls back on climatological baselines and struggles with monthly deviations.

The temporal validation adds an important nuance. Under temporal holdout, Tropical *R*^2^ rises to 0.693—far above the LOCO value of 0.142. This confirms that the LOCO difficulty is a cross-zone regime problem, not a model failure: given Tropical training data, the model predicts Tropical PM_2.5_ well. What it cannot do is *extrapolate* the relationship learned in other zones to the Tropical regime.

The reverse pattern holds at high latitudes. Boreal and Polar zones degrade substantially under temporal validation (*R*^2^ = 0.047 and −0.913, respectively), where they had moderate LOCO skill (*R*^2^ = 0.420 and 0.329). This reflects non-stationary conditions in Arctic and sub-Arctic environments during 2022–2023: accelerated high-latitude warming means the 2019–2021 training period is a poor guide to 2022–2023 surface and meteorological conditions [14,15]. For operational use in polar regions, periodic retraining would be essential.

Better tropical performance will not come from more sophisticated algorithms. Higher-resolution meteorology (ERA5 daily or sub-daily), explicit gridded emissions (EDGAR, CEDS, fire inventories) and chemical-transport information are what is needed [3,26,30]. The performance ceiling here is set by the features, not the algorithm.

### Implications for pollution monitoring and research design

These findings carry a few practical messages for researchers and agencies working on reanalysis-based pollution assessment.

First, reporting a single global skill metric for a model applied across climate zones is misleading. The fourfold spread in *R*^2^ between Subtropical (0.604) and Tropical (0.142) means that a global mean hides the fact that the model is useful in some regions and essentially uninformative in others. Zone- or region-specific performance tables should be standard reporting practice.

Second, the dominance of geographic proxies over meteorological variables is not just a model curiosity—it points to what is missing from the feature set. A model that relies on latitude and longitude to encode emission patterns is implicitly signalling that explicit emission inventories would improve it. Future studies should treat the SHAP geographic fraction as an upper bound on the improvement available from adding emission data.

Third, the ensemble stacking result is a practical warning. Combining three gradient-boosting models with a linear meta-learner made performance worse (*R*^2^ dropped from 0.367 to 0.278), not better, because the base models make correlated errors. For resource-constrained teams, careful tuning of a single model is more valuable than building a stack of similar architectures.

Fourth, the IDW comparison confirms that LightGBM’s skill (*R*^2^ = 0.367) is not just spatial interpolation in disguise. IDW, which uses only location, reaches *R*^2^ = 0.185—the ML model adds a further 0.182 *R*^2^ points by learning how meteorology and geography interact across zone boundaries.

### Implications for hazardous-materials and exposure studies

From the perspective of *hazardous materials* science and environmental exposure research, three points stand out:

1. **Climate-aware uncertainty reporting:** Global PM_2.5_ models used in exposure studies or hazard maps should report zone-specific uncertainty alongside global metrics. Tropical and equatorial populations, where current reanalysis skill is weakest, carry the greatest uncertainty in any downstream exposure estimate derived from models like this one.
2. **Emission-first feature design:** Since geographic proxies dominate meteorology in predictive importance across all zones, future PM_2.5_ assessment models should prioritise incorporating gridded emission inventories, especially in lower-income regions where both monitoring networks and emission data are sparse [3,30].
3. **Explainable AI as a model audit tool:** SHAP analysis revealed that the model was leaning heavily on location even after zone-specific tuning, which motivated the ablation experiment and the spatial baseline comparison. Using explainability tools as part of the model development process—not just as a post-hoc reporting step—is a practical way to catch and quantify this kind of structural reliance [28,29].

## Conclusion

This study shows that a compact feature set—geography, time and MERRA-2 meteorology—combined with a zone-tuned LightGBM model can give a useful benchmark for reanalysis-based PM_2.5_ assessment, particularly in subtropical and mid-latitude regions. The global LOCO *R*^2^ of 0.367, achieved with nested hyperparameter tuning to prevent data leakage, represents a meaningful lower bound on what a meteorology-only, data-driven approach can do across fundamentally different climate regimes. Just as importantly, the framework itself is the contribution: climate-stratified LOCO validation, nested-CV tuning, SHAP explainability, geographic ablation and spatial baseline comparison are all implemented in open, version-controlled scripts archived on Zenodo and GitHub. Other researchers can reproduce these results exactly, benchmark new methods against them or adapt the pipeline to other pollutants, reanalyses or spatial scales.

The results also draw clear lines around what this kind of model cannot do. Climate regime is a hard constraint on predictability: the same predictors and algorithm give *R*^2^ = 0.604 in the subtropics and *R*^2^ = 0.142 in the tropics. The ablation experiment shows why—without latitude and longitude, mean *R*^2^ drops from 0.367 to −0.099, confirming that geographic coordinates carry information the meteorological features simply do not contain. The spatial baseline comparison shows that LightGBM is not just doing spatial interpolation: IDW reaches *R*^2^ = 0.185 using location alone, while the full ML model reaches 0.367 by learning how meteorology and geography interact across zone boundaries. And ensemble stacking with three tree-based models makes things worse (*R*^2^ drops to 0.278), which is a diagnostic rather than a failure: a well-tuned single model has largely exhausted the predictable signal in this feature set, and further gains need new input data, not more complex architectures.

These findings have a few practical messages for teams building reanalysis-based assessment tools. Reporting a single global skill metric is not enough—zone- or region-specific performance should be standard, because the fourfold spread in *R*^2^ across zones means the model is useful in some regions and close to uninformative in others. In regions where skill is low, particularly tropical areas with episodic biomass burning and complex convection, the model’s output should be treated as a rough screening estimate rather than a reliable quantitative prediction. Complementing it with targeted ground monitoring and emission inventories is the right next step, not more sophisticated stacking of the same predictors.

Future work should therefore focus on:

1. **ERA5 integration:** Daily or sub-daily ERA5 meteorology would better capture convective and synoptic-scale drivers, particularly in the tropics where monthly averages miss most of the relevant variability.
2. **Emission inventories:** Adding gridded anthropogenic and fire emissions (e.g., EDGAR, CEDS, GFED) [49,50] directly into the feature space would let the model learn from explicit source information rather than inferring it indirectly from coordinates.
3. **Multi-source satellite fusion:** Combining AOD retrievals from MODIS, MISR, VIIRS and Sentinel-5P would reduce retrieval gaps in cloudy or optically challenging environments, particularly in the deep tropics.
4. **Physics-informed and hybrid models:** Deep-learning or hybrid architectures that embed physical constraints could improve performance while preserving the interpretability that makes these models useful for diagnostics [28].
5. **Ground-truth calibration:** Systematic comparison against regulatory monitoring networks would quantify and correct biases in reanalysis-based estimates, a necessary step before using outputs like these in any downstream exposure or health application.

Tropical regions present the sharpest need for these advances. The model currently struggles most where data are sparsest and pollution burdens are often highest. For those regions, climate-stratified validation and explainability diagnostics are not optional add-ons—they are the only honest way to communicate what the model actually knows and where its estimates should not be trusted.

## Supporting information

S1 Fig Cross-model SHAP consistency comparison. Comparison of SHAP feature importance between zone-tuned LightGBM and zone-tuned XGBoost on the same subtropical held-out test set, confirming that geographic dominance is consistent across architecturally different gradient-boosting models.
(TIF)
